# Carpal Tunnel Syndrome Secondary to Fibrolipomatous Hamartoma of the Median Nerve

**DOI:** 10.7759/cureus.15363

**Published:** 2021-06-01

**Authors:** Christopher R Michel, Christopher Dijanic, Mark Woernle, Justin Fernicola, Jamie Grossman

**Affiliations:** 1 Orthopedic Surgery, Monmouth Medical Center, Long Branch, USA

**Keywords:** fibrolipomatous hamartoma, median nerve, carpal tunnel, carpal tunnel release, hamartoma, orthopedic surgery, hand surgery, plastic surgery, klippel trenaunay syndrome, macrodactyly

## Abstract

Fibrolipomatous hamartoma (FLH) is a rare, benign neoplasm that affects the median nerve predominantly and can present with compressive symptoms. MRI can be used to diagnose this condition without the need for a nerve biopsy. While no definitive treatment has been described, open carpal tunnel release for nerve decompression is currently the standard of care to alleviate compressive neuropathies of the median nerve. In this report, we describe a case of FLH diagnosed via MRI in which the patient’s symptoms responded to open carpal tunnel release.

## Introduction

Fibrolipomatous hamartoma (FLH) is a rare, benign neoplasm that is derived from fibroadipose tissue, which infiltrates the epineurium and perineurium. While this pathology can occur anywhere in the body, the most common site according to the literature is the median nerve. Other sites that have been reported include the ulnar nerve, brachial plexus, and, less commonly, nerves in the lower extremities. It has been associated with several other conditions, including macrodactyly. While nerve biopsy is the gold standard for diagnosis, several findings on MRI are pathognomonic. The treatment for FLH of the median nerve is open carpal tunnel release, although the endoscopic approach has been used. Recurrence is rare. 

## Case presentation

**Figure 1:**
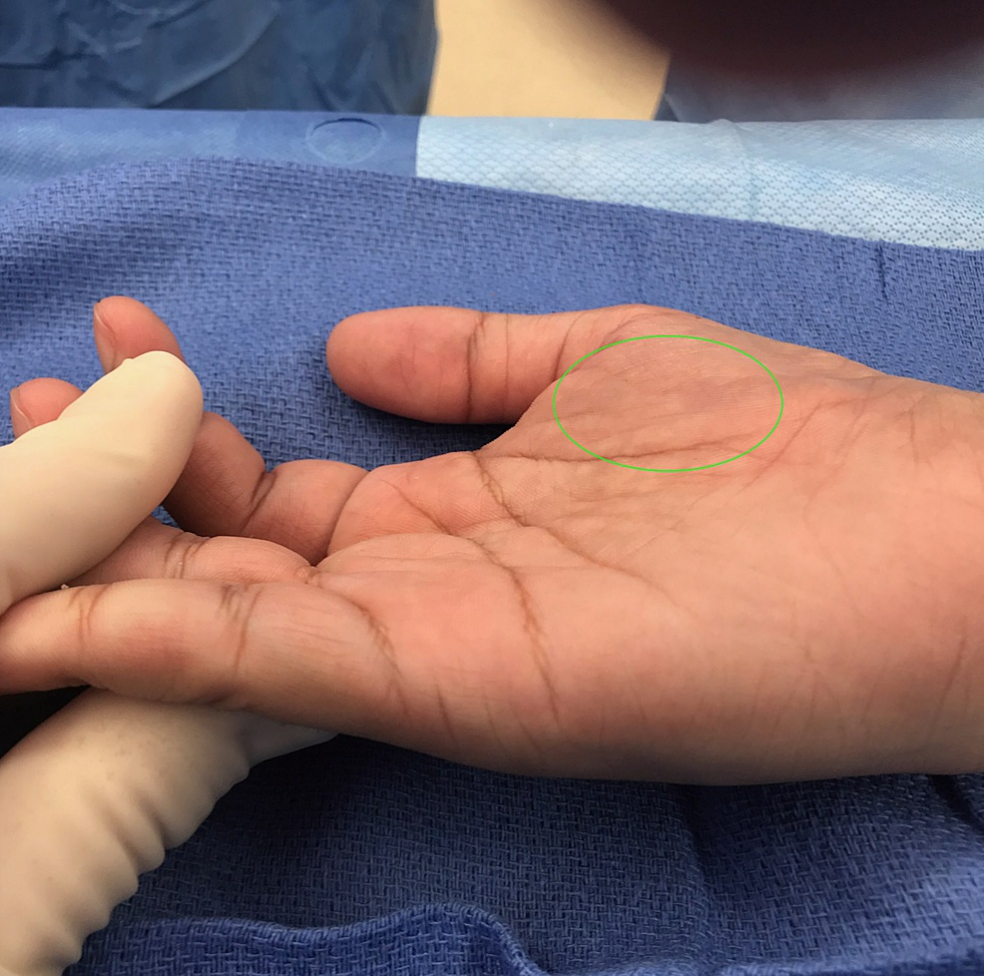
Affected hand of the patient showing severe thenar atrophy identified with the green oval

A 26-year-old right-hand dominant male presented with a lump on his right wrist. Physical examination was significant for thenar atrophy, as shown in Figure 1, and a soft palpable mass on the volar aspect of the wrist. 

**Figure 2:**
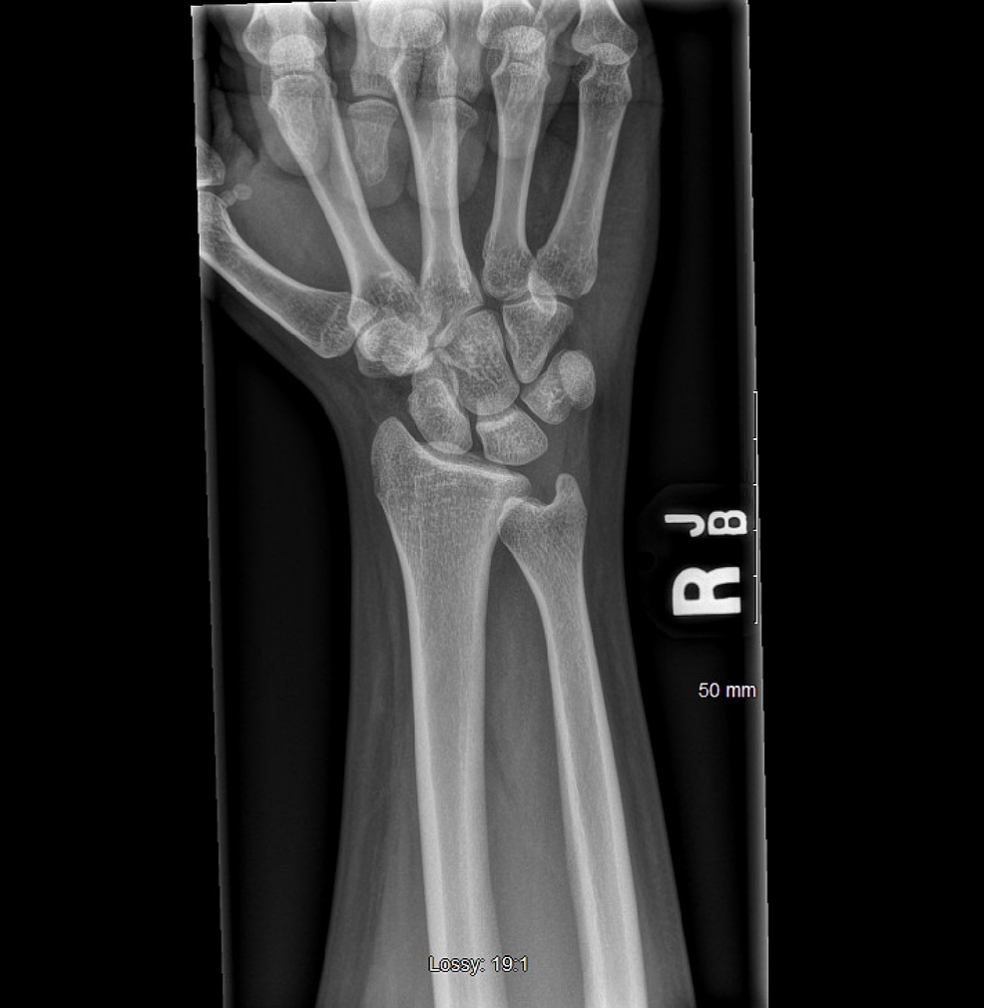
Anteroposterior (AP) X-ray of the patient's right wrist demonstrating normal findings

**Figure 3:**
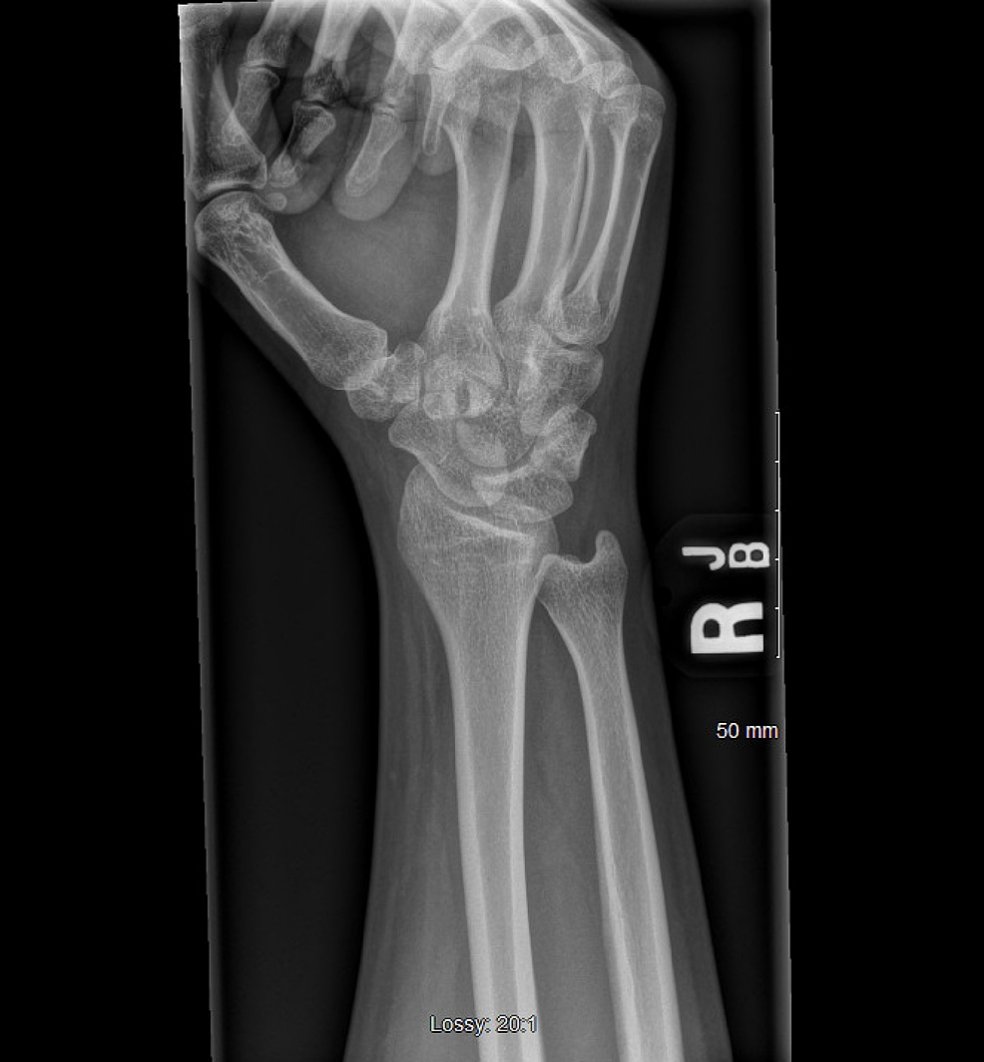
Oblique X-ray of the patient's right wrist demonstrating normal findings

**Figure 4:**
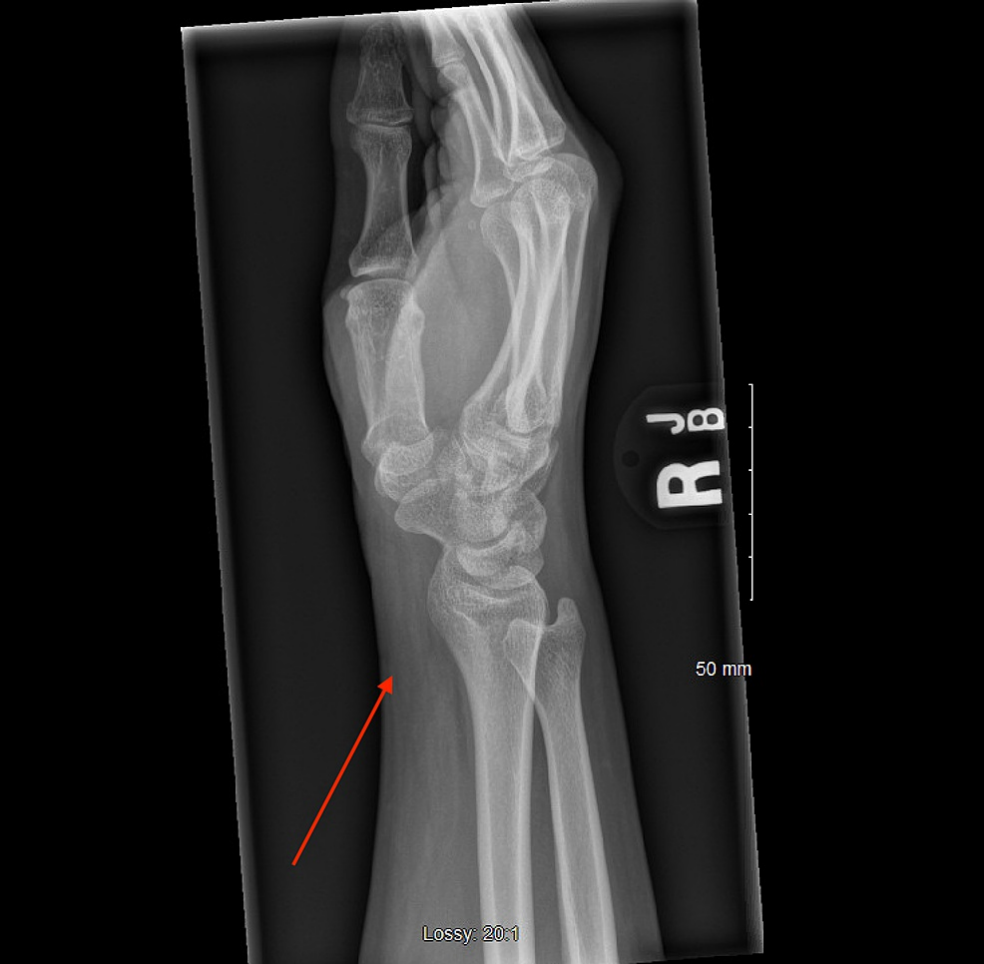
Lateral X-ray of patient's right wrist demonstrating moderate volar soft tissue swelling

**Figure 5:**
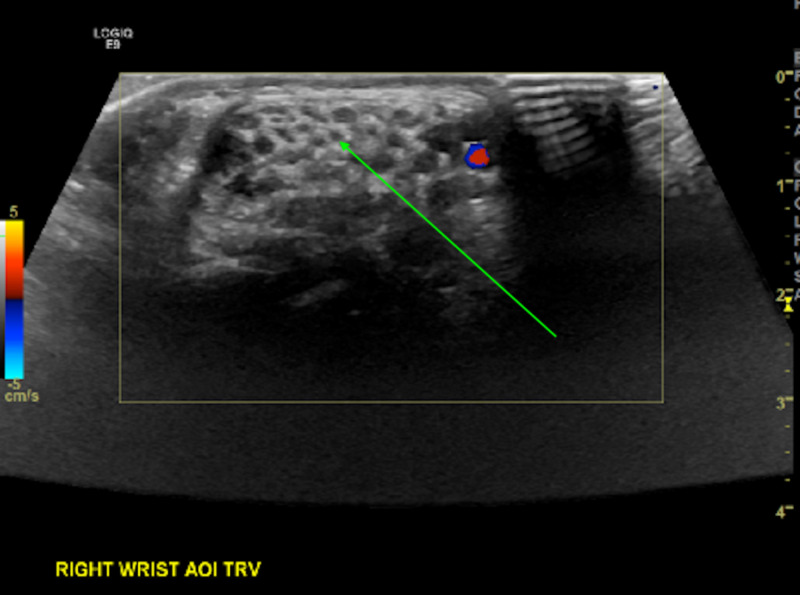
Ultrasound appearance of patient's right wrist showing echogenic fatty tissue interspersed with hypoechoic 'coaxial cabling' identified by the green arrow

**Figure 6:**
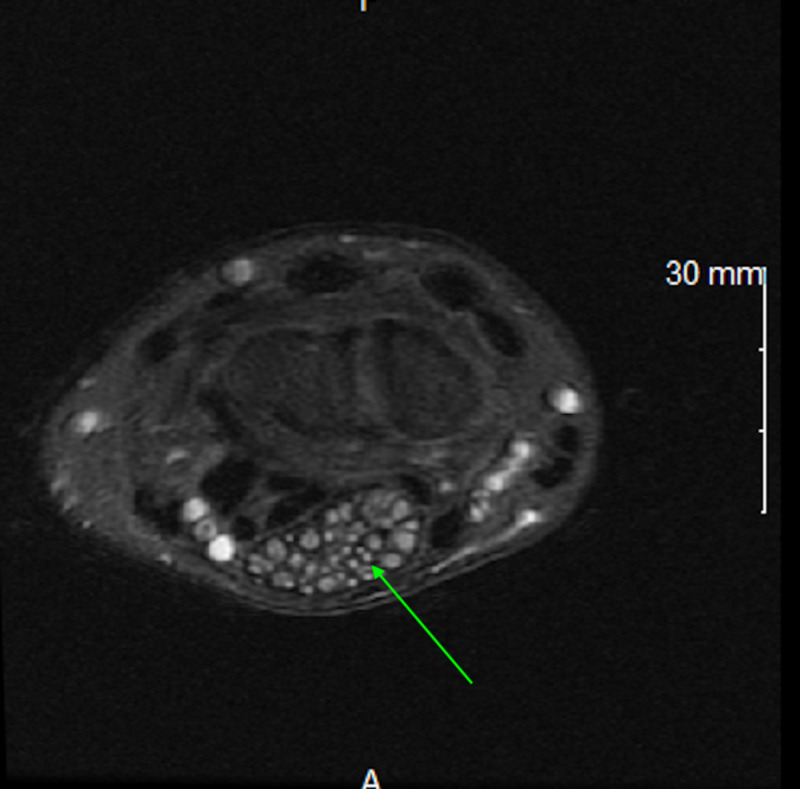
Axial T1 weighted MRI appearance of the right wrist shows 'coaxial cable' appearance in the region of median nerve identified by the green arrow

**Figure 7:**
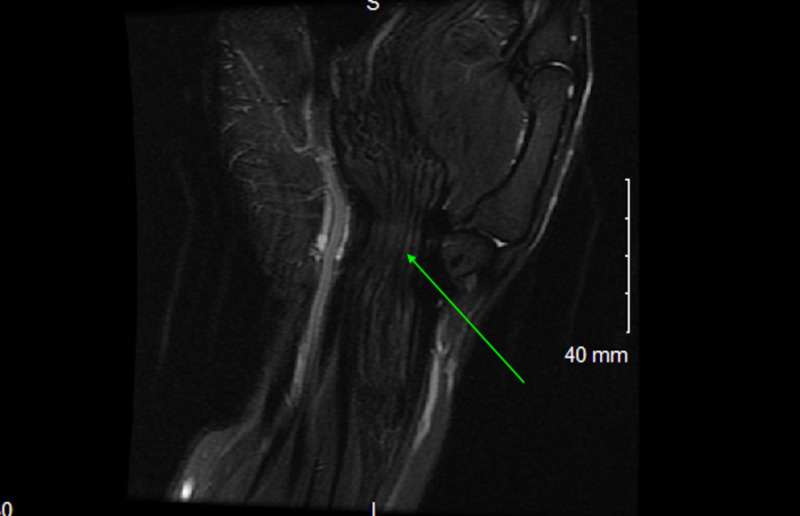
Coronal T1 weighted imaging of right wrist shows 'spaghetti-like' appearance in the region of median nerve identified by the green arrow

X-ray imaging was done to evaluate the mass, which showed soft tissue swelling (Figures 2-4). Ultrasound and MRI were subsequently ordered to evaluate the mass. Ultrasound was significant for non-specific fatty proliferation (Figure 5), while MRI showed fibrofatty proliferation, causing bowing of the flexor retinaculum secondary to mass effect. In addition, a 'coaxial cable' appearance was seen on axial T1 weighted images (Figure 6), while a spaghetti-like appearance was seen on coronal T1 weighted images (Figure 7).

**Figure 8:**
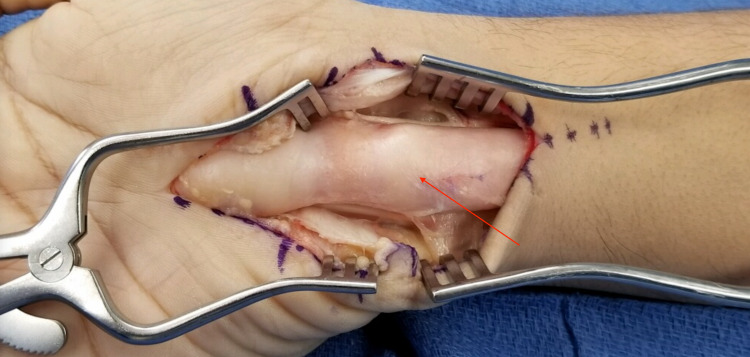
Intraoperative appearance of the fibrofatty mass, which appeared yellow in color

Based on the MRI findings, the patient was diagnosed with right carpal tunnel syndrome secondary to FLH of the median nerve. The treatment of choice was a right open carpal tunnel release. On gross examination, the median nerve appeared enlarged and yellow in appearance (Figure 8). The decision was made not to resect the mass. The patient’s symptoms subsequently resolved. 

## Discussion

FLH is a pathology that was first described by Mason in 1953 as a rare, benign neoplasm that involves peripheral nerves and their branches [1,2]. Over time, this pathology has been described by many other names, including lipofibromatous hamartoma, lipofibroma, fibrofatty proliferation of nerve, intraneural lipoma, neurofibroma, and nerve lipoma. This growth is derived from the fibroadipose tissue within the nerve sheath. This tissue infiltrates the epineurium and perineurium [3]. Over time, this can lead to fusiform enlargement of the nerve causing compressive neuropathies. The median nerve is affected in over 80% of cases [4]; however, there have been reports of involvement of nerves at other peripheral sites, including the radial, ulnar, sciatic, peroneal, plantar, and digital nerves. FLH most commonly occurs in the first three decades of life, and Caucasians are predisposed compared to other races. In addition, males develop FLH more frequently than females unless macrodactyly is present, in which case, females are more likely to develop FLH than males [2-3].

Hypertrophy of mature fat cells and fibroblasts within the epineurium has been proposed to cause the growth. However, the inciting factor of this hypertrophy is unknown. Some believe the neoplasm is congenital, and the growth of FLH is related to an abnormally developed transverse carpal ligament or flexor retinaculum leading to a reactive process secondary to nerve irritation of microtrauma. This repetitive pressure on the nerve from the carpal ligament may initiate a cascade that ultimately culminates in the growth of FLH [3].

FLH is associated with macrodactyly and Klippel-Trenaunay syndrome. FLH was originally thought to have been associated with neurofibromatosis due to its association with macrodactyly; however, there have been no reported cases in which the patient has a family history of neurofibromatosis. In addition, histology of nerves in patients with neurofibromatosis does not show the fatty infiltration seen in FLH [2]. Other associated conditions include Klippel-Trenaunay syndrome, a disease characterized by the triad of cutaneous capillary malformations, varicose veins, and hypertrophy of bone and soft tissue. FLH is less commonly associated with ectopic soft tissue calcification, fatty muscle infiltration, bony exostoses, subcutaneous lipomas, and vascular tumors. 

Cases of FLH often present themselves as compressive neuropathies accompanied by a slow-growing soft tissue mass that may be palpable. Symptoms related to nerve compression include pain, paresthesias, and decreased sensation. These can be progressive for years. FLH most often occurs unilaterally, although cases of bilateral FLH have been described [4-5]. On physical exam, FLH may present as a soft palpable mass, which may be difficult to distinguish from other masses. The differential diagnosis for a hand mass typically includes ganglion cysts, vascular malformations, traumatic neuromas, schwannomas, neurofibromas, and intraneural lipomas [3,6]. Two-point discrimination is a useful test to assess the infiltration of FLH into the nerve. Phalen and Tinel signs have also been used, although their sensitivity and specificity are unknown [7]. 

Several imaging modalities have been used to evaluate FLH, of which MRI is the most useful. X-ray of FLH displays soft tissue swelling in the area of the nerve, which may be indistinguishable from other causes of hand masses. Ultrasound has also been used due to its low cost and instant results. However, the findings are nonspecific and, in the case of FLH, show a hyperechoic fatty infiltration that contains hypoechoic bands representing neural bundles [8]. The soft tissue detail provided by MRI has been used to successfully localize and diagnose a nerve tumor in the upper extremities in 75% of cases [9]. T1 weighted imaging of FLH in the coronal plane is characterized by thickened nerve fascicles evenly dispersed throughout the nerve sheath interposed by fat. This causes a serpiginous or ‘spaghetti-like’ appearance that has been described as resembling a ‘coaxial cable’ in the axial plane [8]. These pathognomonic findings on MRI reduce the need for a nerve biopsy to diagnose FLH [4]. Electromyography (EMG) and nerve conduction studies have not proven to be useful in diagnosing FLH. On pathologic examination, FLH grossly appears a yellow-colored fusiform mass. Histologically, fibrofatty infiltration of epineurium can be seen compressing the fascicle. The epineurium and perineurium are typically disorganized, enlarged, and distorted by excess mature fat and fibrous tissue [6].

While no definitive treatment has been described, open carpal tunnel release has become the standard of care, with the endoscopic approach occasionally being utilized [4]. Lesion resection is controversial and can result in loss of nerve function. In addition, radical excision and fascicular cable graft have yielded poor results. A more conservative approach with debulking of the fibrofatty sheath tissue has been favored [2]. Both regression and progression of compressive symptoms have been reported after open carpal tunnel release [4]. 

## Conclusions

Fibrolipomatous hamartoma of the median nerve remains a poorly understood pathology due to the scarcity of cases. While management is dictated by patient symptoms, more research is needed to evaluate whether the case of these lesions is genetic or spontaneous. 
